# Selection of Superior Lentil (*Lens esculenta* M.) Genotypes by Assessing Character Association and Genetic Diversity

**DOI:** 10.1155/2014/372405

**Published:** 2014-12-11

**Authors:** U. K. Nath, Santona Rani, M. R. Paul, M. N. Alam, B. Horneburg

**Affiliations:** ^1^Department of Genetics and Plant Breeding, Bangladesh Agricultural University, Mymensingh 2202, Bangladesh; ^2^Bangladesh Sugarcane Research Institute, Ishurdi, Pabna 6620, Bangladesh; ^3^Division of Plant Breeding, Georg-August University, 37075 Göttingen, Germany

## Abstract

Lentil is one of the most important pulse crops in the world as well as in Bangladesh. It is now considered a main component for training and body building practising in first world countries. Yield varies tremendously from year to year and location to location. Therefore, it is very important to find genotypes that perform consistently well even in ecological farming systems without any intercultural operations. Twenty lentil genotypes were tested during the period from November 2010 to March 2011 and from December 2011 to March 2012 with three replicates in each season to determine genetic variability, diversity, characters association, and selection indices for better grain yield. The experiment was conducted at the breeding field of the Department of Genetics and Plant Breeding, Bangladesh Agricultural University, Mymensingh. This study revealed that all the genotypes possess a high amount of genetic diversity. Plant height and 100-grain weight showed significant positive correlation with grain yield plant^−1^ that was also confirmed by path analysis as the highest direct effect on grain yield. The genotypes BM-513 and BM-941 were found to be the best performer in both the seasons and were considered as consistent genotype. The genotypes were grouped into four clusters based on Euclidean distance following Ward's method and RAPD analysis. However, discriminant function analysis revealed a progressive increase in the efficiency of selection and BM-70 ranked as the best followed by the genotypes BM-739, BM-680, BM-185, and BM-513. These genotypes might be recommended for farmers' cultivation in ecological farming in Bangladesh.

## 1. Introduction

Lentil (*Lens esculenta* M.) is one of the most important pulse crops. It is cultivated in many parts of the world covering tropical, subtropical, and temperate regions. Nutritionally lentil is very rich in protein and complementary to any cereal crop including rice. Lentil is known as poor man's meat. The development of genotypes with good, stable yield and higher protein content is important to improve the yield status of lentil. The yield potential of this crop needs to be improved through an effective plant breeding program. The environment has much influence on the productivity of lentil genotypes. Therefore, direct selection for grain yield is often misleading because it is influenced by component characters. Moreover, grain yield depends on a number of yield contributing characters. So, yield along with its contributing characters should be considered in determining the selection criteria for yield improvement. The success of breeding programs also depends upon the amount of genetic variability present in the population and the extent to which the desirable traits are heritable.

It is widely accepted that information on germplasm diversity and genetic relatedness among elite breeding materials is a fundamental element in plant breeding [1]. The present investigation is, therefore, carried out with a view to assess the genetic variability, the relationship between yield and yield contributing characters, and selection indices and their relative efficiencies of yield of lentil at ecological farming.

## 2. Materials and Methods

### 2.1. Plant Materials

16 local breeding lines (LBLs) and 4 genotypes from a German research program [2] were used in this study. Indigenous material was received from the Department of Genetics and Plant Breeding, Bangladesh Agricultural University, Mymensingh. The LBLs were BM-739, BM-70, BM-1227, BM-513, BM-1222, BM-983, BM-848, BM-279, BM-680, BM-593, BM-295, BM-981, BM-185, BM-828, BM-941, and BM-880 and the German lines were Pisarecka Perla, Marmorierte Linse, Gestreifte Linse, and Schwarze Linse.

### 2.2. Experimental Methods

The experiments were conducted during the period from November 2010 to March 2011 and from December 2011 to March 2012 at the breeding field of the Department of Genetics and Plant Breeding, Bangladesh Agricultural University, Mymensingh. The experiment was set up in randomized complete block (RCB) design with three replicates. The plot size was 3 m × 5 m. The distance between two plots was 50 cm and the distance between two blocks was 100 cm. The 20 lentil genotypes were sown in the field on November 16, 2010, and on December 06, 2011, respectively. The seeds were sown in line sowing with 20 cm in seed to seed and 0.50 cm in line to line spacing. Therefore, 150 plants were grown in each plot. The land was occupied with rice cultivation in the previous crop season. Plants were allowed to grow in ecological farming without any intercultural operations and any additional nutrients supplement. Genotypes matured at different times. Harvesting was completed by March 18 to March 27 in 2011 and by March 15 to March 25 in 2012. Data on the plant characters were recorded on individual plant basis from 5 randomly selected plants per plot. DNA was extracted from leaves of 25-day-old plants of lentil genotypes by the CTAB method. Extracted DNA quality was confirmed. RAPD markers were amplified by polymerase chain reaction (PCR) and electrophoresis of amplified products was documented and polymorphic bands were scored for analyzing genetic diversity.

### 2.3. Statistical Analysis

Analysis of variance was calculated by using PLABSTAT Version 2N [3] software using the following statistical model:

(1) $$\begin{array}{c} Y_{ijk}=\mu +g_{i}+y_{j}+r_{k}+{gy}_{ij}+\varepsilon_{ijk}, \end{array}$$

where *Y*_*ijk*_ was observation of genotype *i* in year *j* and replicates *k*, *g*_*i*_, *y*_*j*_, and *r*_*k*_ were effects of genotype *i*, year *j*, and replicate *k*, respectively, and *gy*_*ij*_ and *ε*_*ijk*_ were the interaction between genotypes and year and residual error of genotype *i* in year *j* and replicate *k*. Multiple mean comparisons were made with Fisher's least significant difference (LSD) procedure using Stat Graphics Plus for Windows 3.0 (Statistical Graphics Corp., Rockville, USA). Heritability was estimated in broad sense by the following formula suggested [4, 5]:

(2) $$\begin{array}{c} \text{Heritability}\left(h_{b}^{2}\right)=\frac{\sigma_{g}^{2}}{\sigma_{p}^{2}}\times 100, \end{array}$$

where *σ*_*g*_^2^ is genotypic variance and *σ*_*p*_^2^ is phenotypic variance.

Direct and indirect effects of the path coefficient analyses were calculated as described by Lynch and Walsh [6].

To determine the direct effect, square matrices of the correlation coefficients between independent traits in all possible pairs were inverted and multiplied by the correlation coefficients between the independent and dependent traits. Path coefficient was performed considering yield as effect variable and others were causal variables. Euclidean Ward's method was applied to study genetic diversity. This method attempts to minimize the sum of squares of any two (hypothetical) clusters that can be formed at each step [7]. Selection index was constructed using the methods developed by Smith [8] based on the discriminate function analysis following the simultaneous selection model described [9]. RAPD data were analysed with Nei's [10] gene diversity and Nei's [11] genetic distance and constructed a UPGMA (unweighted pair group method of arithmetic means) dendrogram among populations using a computer programme, POPGENE Version 1.31 [12].

## 3. Results and Discussion

**Figure 1:**
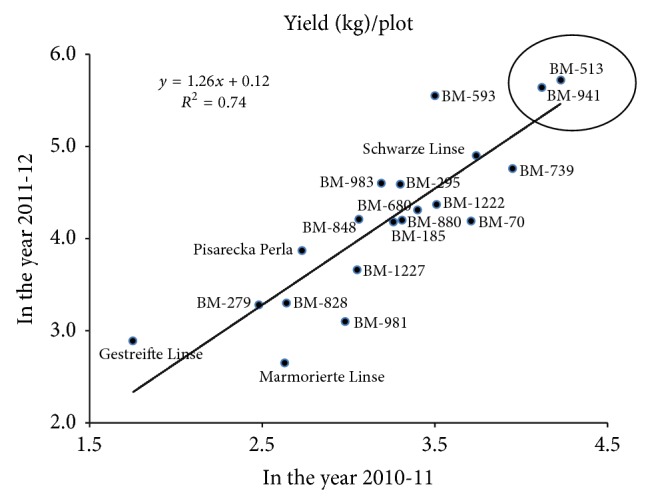
Yield of 20 lentil genotypes in ecological farming in two growing seasons (2010-11 and 2011-12).

The analysis of variance revealed that significant variability existed among the genotypes for their performance except yield per plant. However, genotype and year interaction showed significant variation even for yield per plant, indicating that the growing season had large influence on yield in ecological farming (Table 1). Grain yield was positively correlated with plant height (*r* = 0.44^*^) and 100-grain weight (*r* = 0.56^*^); this was also confirmed by the direct effect of path coefficient analysis (Table 2). Correlation and path analysis revealed that plant height and 100-grain weight had a major contribution to increasing yield of lentil in ecological farming. Therefore, it is necessary to give more attention to these two traits for the further improvement of lentil yield for ecological farming. Rakesh et al. [13] and Hamdi et al. [14] reported a positive and significant association between seed yield number of pods, number of seeds per pod, plant height, and number of branches plant^−1^. A negative but nonsignificant correlation was observed between grain yield plant^−1^ and seeds pod^−1^ (*r* = −0.43) which is controversial to the general phenomena of the yield attributes. However, 100-grain weight showed significant and positive correlation with primary branches plant^−1^ (*r* = 0.44^*^), pods plant^−1^ (*r* = 0.65^**^), and seeds pod^−1^ (*r* = −0.49^*^). These results revealed that the pod that set at primary branches produced comparatively larger seeds. Anita et al. [15] had also observed significant positive correlation between plant height and yield plant^−1^. However, Rathi [16] observed a significant negative association between plant height and 100-grain weight. The genotypes that exhibited comparatively higher yield in both the seasons (marked as circle in scattered diagram; Figure 1) could be concluded as stable in performance. Among the exotic (German) genotypes, only Schwarze Linse performed at satisfactory level regarding yield/plot 3.74 and 4.9 kg in the years 2010-11 and 2011-12, respectively. However, yield of the other 3 German genotypes did not reach a satisfactory level compared to local collection (Figure 1).

**Figure 2:**
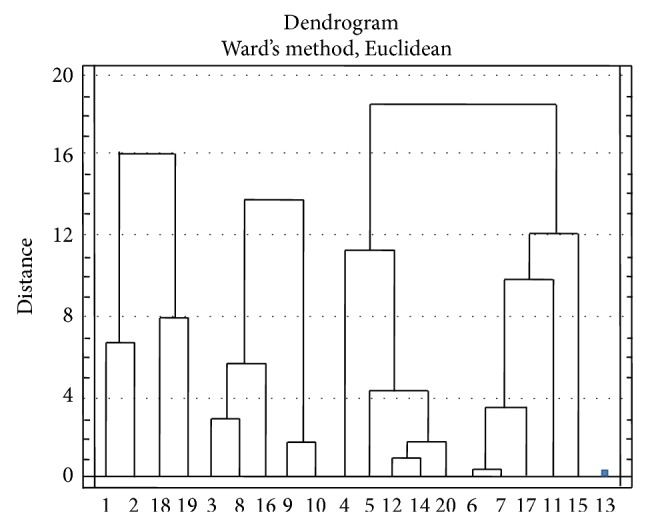
Dendrogram based on Euclidean distance, summarizing the data on differentiation among 20 lentil genotypes according to Euclidean Ward's method (1: BM-739, 2: BM-70, 3: BM-1227, 4: BM-513, 5: BM-1222, 6: BM-983, 7: BM-848, 8: BM-279, 9: Marmorierte Linse, 10: Gestreifte Linse, 11: BM-593, 12: BM-295, 13: BM-981, 14: BM-185, 15: Pisarecka Perla, 16: BM-828, 17: Schwarze Linse, 18: BM-680, 19: BM-941, and 20: BM-880).

Using Euclidean distance following Ward's method, the 20 lentil genotypes were grouped into four distinct clusters (Table 3). Clusters III and IV contained 10 and 1 genotypes and I and II contained 4 and 5 genotypes, respectively. The members of cluster I were BM-739, BM-70, BM-680, and BM-941. The members of cluster II were BM-1227, BM-279, BM-828, Marmorierte Linse, and Gestreifte Linse. Cluster III consisted of genotypes BM-513, BM-1222, BM-295, BM-185, BM-880, BM-983, BM-848, Schwarze Linse, BM-593, and Pisarecka Perla; only genotype BM-981 formed cluster IV. In cluster I, the genotypes BM-739 and BM-70 together and BM-680 and BM-941 together were forming two separate subclusters and the distance of cluster I was from 0 to 16. On the other hand, in cluster II, genotypes BM-1227, BM-279, and BM-828 were forming subcluster with genotypes Marmorierte Linse and Gestreifte Linse and the distance of cluster II was 0–13.80. The largest cluster, cluster III, contained two separate subclusters; the members of subcluster (a) were BM-513, BM-1222, BM-295, BM-185, and BM-880 and those of subcluster (b) were BM-983, BM-848, Schwarze Linse, BM-583, and Pisarecka Perla. The distance of cluster III was 0–18.50 (Figure 2) indicating the existence of considerable genetic diversity in the germplasm. A similar result was reported by Chauhan et al. [17] who reported that the nonhierarchical Euclidean cluster analysis grouped the 60 genotypes of lentil into 6 distinct clusters. Therefore, it could be concluded that breeders might use the genotypes belonging to different clusters for hybridization to get maximum heterosis.

A selection index was constructed to identify suitable genotypes among the 20 genotypes of lentil following simultaneous selection model, considering six yield attributes. Genotype BM-70 possessed the highest selection index (424.94) and ranked as the best followed by the genotypes BM-739, BM-680, BM-185, and BM-513 with 331.79, 267.01, 256.83, and 254.41, respectively (Table 5). The genotype Pisarecka Perla was the worst possessing the lowest selection score (108.68) followed by BM-828 (124.14) and Marmorierte Linse (157.17). The expected genetic gain (Δ*G*) was 398.44 at 5% selection intensity; that is, 2-3 highest scoring genotypes, for example, BM-70 and BM-739, might be recommended for farmers' cultivation to increase yield in ecological farming. The level of polymorphism (100%) indicated the effectiveness of RAPD technique in detecting substantial polymorphisms and diversity among different lentil genotypes (Table 4). The banding patterns of lentil genotypes using primers GLA-10, OPG-5, and OPG-8 are shown in Figure 2. Pairwise comparisons of Nei's [11] genetic distance (GD) between lentil genotypes were computed from combined data of three primers; values ranged from 0.1112 to 1.3350. Comparatively higher genetic distance was observed between the genotypes BM-739 versus BM-1222, BM-739 versus BM-295, BM-739 versus BM-828, and BM-70 versus BM-279. The lowest genetic distance 0.1112 was observed between the genotypes BM-1227 versus BM-1222, BM-279 versus BM-185, and BM-185 versus BM-680. Genetic identity between genotypes was found for the 3 primers, ranging from 0.8947 to 0.2632. The highest genetic identity (0.8947) was found in BM-1222 versus BM-70, BM-279 versus BM-185, and BM-185 versus BM-680. The lowest genetic identity (0.2632) was observed in BM-739 versus BM-1222, BM-739 versus BM-295, BM-739 versus BM-828, and BM-70 versus BM-279.

A dendrogram was constructed based on Nei's [11] genetic distance following the unweighted pair group method of arithmetic means (UPGMA). The lentil genotypes were grouped into two main clusters A and B (Figure 2). Genotypes BM-513, BM-828, BM-185, BM-279, BM-295, BM-680, BM-981, BM-1222, BM-1227, and BM-848 were included in cluster B. Cluster B again divided into two subclusters: B-I and B-II. Genotypes BM-513 and BM-828 formed subcluster B-II and genetic relationship was present between them. Again, among the genotypes of subcluster B-I, BM-848, BM-1227, and BM-1222 formed sub-subcluster 1 and BM-981, BM-680, BM-295, BM-279, and BM-185 belonged to sub-subcluster 2. In these clusters, genetic variation was present among these genotypes. Genotypes belonging to cluster A were BM-739, BM-983, BM-70, BM-941, BM-593, and BM-880. The genotypes of cluster A again divided into two subclusters *a*_1_ and *a*_2_. Subcluster *a*_1_ consisted of genotypes BM-70, BM-941, BM-593, and BM-880. Subcluster *a*_2_ formed by two genotypes BM-739 and BM-983; genetic relationship was present between subclusters. Genotypic variations based on RAPD markers indicated that genotypes belonging to different clusters depend on their genetic components themselves, but not at geographical origin at all. Therefore, it could be concluded that for further research programme, especially for hybridization, genotypes could be selected from different clusters for getting maximum heterosis regarding yield. Rana et al. [18] observed UPGMA cluster analysis for the combined data of RAPD and STMS revealed two broad clusters—Cluster I with three landraces and Cluster II containing all remaining landraces and cultivars except Precoz. Precoz was found to be the most distinct in individual as well as combined analysis.

## 4. Conclusion

Two experiments were conducted with an objective to assess the genotype-environment interaction among 20 lentil genotypes and the performance of these lentil genotypes, their association, morphological diversity, selection index, and molecular variability for improvement of yield of lentil. Plant height, pods plant^−1^, seeds pod^−1^, and grain yield plant^−1^ were significantly influenced by genotype, environment, and their interaction. The highest direct effect of plant height and 100-grain weight ultimately gave a significant positive correlation among all the characters and these characters contributed maximum to grain yield. From the study, it may be concluded that, among the lentil genotypes, BM-739, BM-1222, BM-70, BM-295, BM-279, and BM-828 contained the highest number and proportion of polymorphic loci and gene diversity. This study indicated that BM-739, BM-1222, BM-70, BM-295, BM-279, and BM-828 contained the highest genetic variations and BM-1227, BM-1222, BM-279, BM-680, and BM-185 contained the lowest genetic variations. High genetic variability and significant genetic differentiation between genotypes indicated potential genetic resources of lentil that can be used for the selection of superior genotypes for commercial cultivation at growers' level as well as for breeding new genotypes of lentil.

## Figures and Tables

**Table 1:** Analysis of variance for six yield contributing characters in 20 lentil genotypes and their interaction in two growing seasons (2010-11 and 2011-12).

Items	d.f.	Plant height	Primary branches plant^−1^	Pods plant^−1^	Seeds pod^−1^	100-grain weight	Yield plant^−1^
Genotype (g)	19	114.60^**^	0.96^**^	29660.29^**^	0.66^**^	0.08^**^	2.52
Year (y)	1	477.20^*^	1.40	36283.04^**^	0.83^**^	0.01^**^	39.51^**^
g × y interaction	19	6.31^**^	0.03	161.78^**^	0.08^**^	0.003	1.49^**^
Error	76	2.29	0.02	57.18	0.001	0.001	0.06

^**^Significant at 0.01 probability.

**Table 2:** Associations of different yield contributing characters and their direct effect on yield in ecological farming in lentil in two growing seasons (2010-11 and 2011-12).

Characters	Primary branches plant^−1^	Pods plant^−1^	Seeds pod^−1^	100-grain weight	Yield plant^−1^	Direct effect on yield
Plant height	0.47^*^	0.17	−0.20	0.39	0.44^*^	0.27
Primary branches plant^−1^		0.01	−0.07	0.44^*^	0.28	0.01
Pods plant^−1^			−0.48^*^	0.65^**^	0.42	0.11
Seeds pod^−1^				−0.49^*^	−0.43	−0.19
100-grain weight					0.56^*^	0.28

^∗,∗∗^Significant at 0.05 and 0.01 probability, respectively.

**Table 3:** Clustering patterns of 20 genotypes of lentil based on Euclidean distance following Ward's method and the genotypes included in respective clusters.

Cluster	Number of genotypes	Percent contribution to diversity	Name of genotypes present in each cluster
I	4	20	BM-739, BM-70, BM-680, and BM-941
II	5	25	BM-1227, BM-279, Marmorierte Linse, Gestreifte Linse, and BM-828
III	10	50	BM-513, BM-1222, BM-983, BM-848, BM-593, BM-295, BM-185, Pisarecka Perla, Schwarze Linse, and BM-880
IV	1	5	BM-981

**Table 4:** RAPD primers with corresponding bands score and their size range together with polymorphic bands observed in 16 lentil genotypes.

Primer codes	Sequences (5′-3′)	Total number of bands scored	Size ranges (bp)	Number of polymorphic bands	Proportion of polymorphic loci (%)
GLA-10	GTGATCGCAG	7	180–1000	7	100
OPG-5	GTGATCGCAG	6	300–700	6	100
OPG-8	CCGCCCAAAC	6	200–600	6	100

Total		19		19	
Average		6.33		6.33	100

**Table 5:** Selection score rank and expected genetic gain of 20 genotypes of lentil considering six characters (plant height, primary branches plant^−1^, pods plant^−1^, seeds pod^−1^, 100-grain weight, and grain yield plant^−1^).

Serial number	Genotypes	Selection score	Rank	Expected genetic gain
1	BM-739	331.79	2	398.44
2	BM-70	424.94	1	
3	BM-1227	199.85	11	
4	BM-513	254.41	5	
5	BM-1222	222.58	9	
6	BM-983	184.87	14	
7	BM-848	181.11	15	
8	BM-279	189.24	13	
9	Marmorierte Linse	157.17	18	
10	Gestreifte Linse	168.82	17	
11	BM-593	253.62	6	
12	BM-295	234.45	8	
13	BM-981	172.67	16	
14	BM-185	256.83	4	
15	Pisarecka Perla	108.68	20	
16	BM-828	124.14	19	
17	Schwarze Linse	197.51	12	
18	BM-680	267.01	3	
19	BM-941	211.31	10	
20	BM-880	251.33	7	
